# Forecasting Model Based on Lifestyle Risk and Health Factors to Predict COVID-19 Severity

**DOI:** 10.3390/ijerph191912538

**Published:** 2022-10-01

**Authors:** Najada Firza, Alfonso Monaco

**Affiliations:** 1 Dipartimento di Economia e Finanza, Università degli Studi di Bari “Aldo Moro”, Largo Abbazia S. Scolastica, 70124 Bari, Italy; 2 Faculty of Economic, Political and Social Sciences, Catholic University Our Lady of Good Counsel, Rr. Dritan Hoxha 123, Laprake, 1031 Tirana, Albania; 3 Dipartimento Interateneo di Fisica M. Merlin, Università degli Studi di Bari Aldo Moro, Via G. Amendola 173, 70125 Bari, Italy; 4 Istituto Nazionale di Fisica Nucleare (INFN), Sezione di Bari, 70124 Bari, Italy

**Keywords:** COVID-19, machine learning, random forests, forecasting models, generalized linear model, support vector machine, feature selection, lifestyle risk factor, flu, vaccination

## Abstract

The COVID-19 pandemic has now spread worldwide, becoming a real global health emergency. The main goal of this work is to present a framework for studying the impact of COVID-19 on Italian territory during the first year of the pandemic. Our study was based on different kinds of health features and lifestyle risk factors and exploited the capabilities of machine learning techniques. Furthermore, we verified through our model how these factors influenced the severity of the pandemics. Using publicly available datasets provided by the Italian Civil Protection, Italian Ministry of Health and Italian National Statistical Institute, we cross-validated the regression performance of a Random Forest model over 21 Italian regions. The robustness of the predictions was assessed by comparison with two other state-of-the-art regression tools. Our results showed that the proposed models reached a good agreement with data. We found that the features strongly associated with the severity of COVID-19 in Italy are the people aged over 65 flu vaccinated (24.6%) together with individual lifestyle behaviors. These findings could shed more light on the clinical and physiological aspects of the disease.

## 1. Introduction

On 11 March 2020, the World Health Organization (WHO, Geneva, Switzerland) declared a global COVID-19 pandemic after a succession of epidemiological events in a rapid timeline. This unknown disease was reported as pneumonia of unidentified etiology and limited to some cases in the city of Wuhan (China) on 31 December 2019. The Chinese Center for Disease Control and Prevention on 9 January 2020 identified the cause of such pneumonia as a new coronavirus (SARS-CoV-2). On 30 January 2020, the WHO declared the international coronavirus emergency in China. Shortly thereafter, the epidemic turned into a pandemic. After over a year, several clinical and demographic factors affecting the COVID-19 mortality have been thoroughly investigated [1,2], but no working diffusion model has been developed.

Italy became one of the Western countries most affected by the COVID-19 pandemic and was the second country after China with the highest number of positives and deaths during the first phase of the pandemic [3]. In this period, Italy was divided into three different geographical areas based on spread and disease severity [4]. Some regions of Northern Italy were the most affected, with Lombardy as the main outbreak of the pandemic [5]. On the other hand, for the central–southern region, the spread of the pandemic was moderate with the only exception of the provinces of Rome and some provinces of the Marche. These geographical differences remained constant during the first wave. The Italian government has introduced measures to prevent the spread of the virus. These measures were very drastic and relied on the total isolation of the country. However, such restrictive measures seem to have limited the the SARS-CoV-2 outbreak and reduced new cases. At the European level, it was possible to note that the COVID-19 pandemic involved the most urbanized and connected countries in the first phase, showing a marked spread among the most industrialized ones. As for Italy, the problems immediately appeared more articulated and complex. The causes behind the virus severity in Italy can be many and complex even because the circulation of SARS-CoV-2 virus started several months before the first patient was identified [6].

The strong intensity and severe discrepancy of the infection among Italian regions has strengthened the hypothesis that factors of a territorial nature, physical and/or social, have influenced the spread of the SARS-CoV-2 virus. These factors appear as clues from which to start in order to understand the vulnerabilities of some territories. In fact, although they do not provide direct answers to why the epidemic took a dramatic drift in Lombardy, they indirectly suggest their importance in favoring contagion or the establishment of situations of high risk of contracting a serious form of the disease. These factors can be classified as social and health characteristics determining the intensity of diffusion such as urbanization and commuting as well as the healthcare system. We took some of these factors to build a model based on machine learning techniques to study the severity of the COVID-19 pandemic in Italy. Furthermore, through feature importance techniques, we quantify how these factors influenced the severity of the pandemics.

Some factors analyzed in this paper have already been linked in some way to the COVID-19 pandemic by previous works. Amato et al. [7] highlighted a correlation between the number of deaths caused by the coronavirus and the level of vaccination coverage against influenza people aged 65 years and older. The research was based on Italian regional data and observed an inversely proportional relationship between the vaccine diffusion, especially in the most fragile sections of the population, and the official number of deaths and infected. A confirmation of these findings is provided by the work published of Zanettini et al. [8] which focused attention on the situation in the USA. Conlon et al. [9] observed that flu vaccination was associated with decreased positive COVID-19 testing, while Wilcox et al. assessed that influenza vaccination was significantly linked with lower likelihood of hospitalization or mortality due to COVID-19 [10]. Gao et al. [11] found that individual lifestyle behaviors and health status could affect the occurrence of COVID-19, while Muhammad et al. [12] reported an association among neurological complications due to COVID-19 and chronic alcohol abuse. Yang et al. [13] showed that patients with allergic rhinitis and asthma have a greater risk of susceptibility to SARS-CoV-2 infection and severe clinical outcomes of disease.

## 2. Materials and Methods

### 2.1. Study Design and Settings

**Figure 1:**
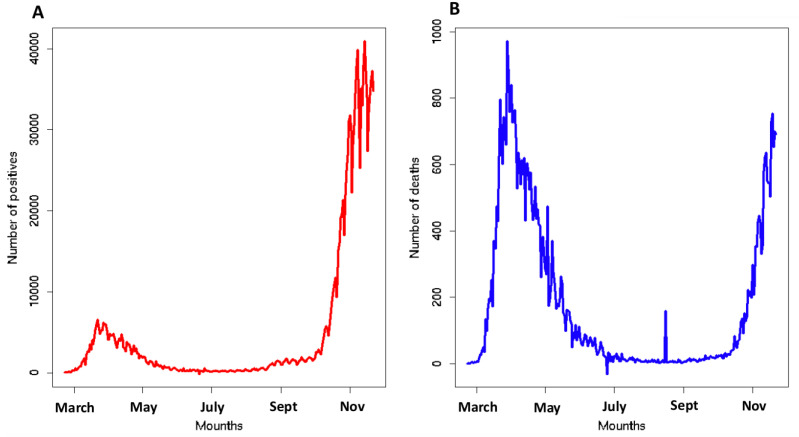
Historical series of positives Panel (**A**) and deaths Panel (**B**) for COVID-19 in Italy. The graph includes the values from 22 February to 22 November 2020.

To build the design of our framework, we considered the number of infected and deaths caused by SARS-CoV-2 from 22 February to 22 November 2020 in Italy (see Figure 1). The high severity requires a deep reflection on the causes of the COVID-19 escalation in Italy. We developed a model to explain SARS-CoV-2 mortality and positivity in 21 Italian regions exploiting three kinds of independent variables: (i) related to people mobility; (ii) related to the incidence of respiratory diseases; and (iii) linked to the individual lifestyle behavior (further details in Section 2.3). We analyzed two more popular ratios to describe the spread of the pandemic: Crude Positivity Rate (CPR) and Crude Mortality Rate (CMR). Our aim was to build a prediction model of CPR and CMR through three different multivariate regressors: Generalized Linear Models, Random Forests and Support Vector Machines. The causes of the spread level of the COVID-19 pandemic can be a lot and range from social factors related (lifestyles or mobility) to environmental and health factors. The complex and multifactorial etiology of COVID-19 cannot be suitably explored by standard techniques and traditional approaches. A model that is able to manage data of a different nature could help to better understand the problem. In this context, techniques based on machine learning paradigms could be of great help thanks to their intrinsic ability to manage multimodal data and represent complex problems. Furthermore, through a feature importance procedure, we established which factors analyzed most influenced our results. We analyzed CPR and CMR up to November 2020 to avoid the confounding effect of the COVID-19 vaccination campaign that started in January 2021.

**Figure 2:**
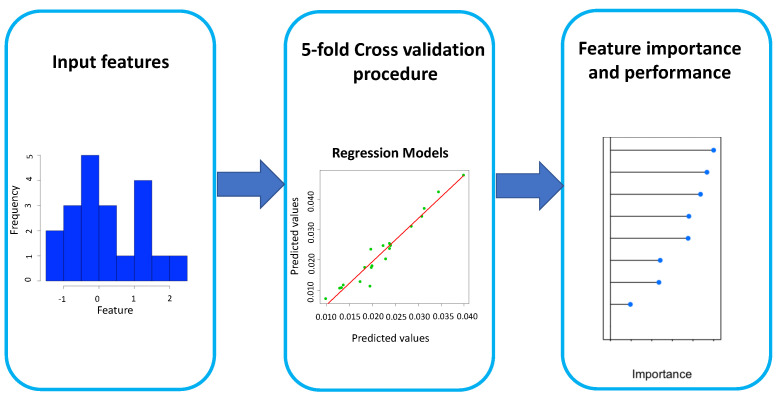
Flowchart of the proposed methodology.

Figure 2 shows the flowchart of our approach. We used 9 lifestyle and health factors as the input features of 3 regression models to provide a quantitative, comprehensive (but not exhaustive) modeling of SARS-CoV-2 pandemics in Italian regions. For each implemented model, we evaluated the feature importance. The different time intervals of features used in the work were decided based on the available datasets, but we believe that the values of the dependent chosen features are quite constant over a wide time interval.

### 2.2. Study Area

The area of our study regarded the whole Italian territory divided into the 21 regions or nomenclature of territorial units for statistics (NUTS 2). For each region, we considered the factors described in the following section.

### 2.3. Data Collection

Epidemiological data for SARS-CoV-2 mortality and positivity of 21 Italian regions were collected from the Italian Civil Protection’s data repository [14]; data relating to social and lifestyle factors were extracted from the Italian Ministry of Health and Italian National Statistical Institute. In our work, the term (i) “positivity” refers to the total number of subjects tested positive on SARS-CoV-2 swabs updated at 22 November 2020, in each Italian region, and (ii) “mortality” refers to the number of people dead because of SARS-CoV-2 updated at 22 November 2020, in each Italian region. We defined the Crude Positivity Rate (CPR) as the ratio between coronavirus positive swabs tests and the total regional population size and Crude Mortality Rate (CMR) as the ratio between dead and the total regional population size [15]. In Table 1, we inserted some information about independent features used in our model.

#### 2.3.1. Machine Learning Approach

We used three multivariate regression algorithms to forecast CPR and CMR and to identify which features were shown to be the most important in the implemented models.

Since there is no guarantee that the variables used in our study are independent and, more importantly, there is no evidence that the relationships between these variables are linear, we rejected here any a priori hypothesis about the data and, therefore, we considered a more general approach also using machine learning algorithms as regressors.

The implemented models are Generalized Linear Models and two machine learning algorithms: Random Forests and Support Vector Machines. The use of three different kinds of models makes the results of our analysis more robust.

#### 2.3.2. Generalized Linear Model

The Generalized Linear Model (GLM) completes in a sense the view of the linear regression model [20]. The linear model hypothesizes that the expected value of the dependent variable *y* is computed as a linear combination of the independent variables *x*. This introduces a limitation of linearity which narrows the practical field of application. Instead, a Generalized Linear Model introduces a linearizing link function which transforms the expectation of the dependent variable [21]. In this way, also non-normal and discrete distributions of *y* can be described by means of this model [22]. Specifically GLM is composed by three components [23]:

1. A random component that specifies the conditional distribution of the dependent variable $y=y_{i},\cdots ,y_{n}$ composed by *n* independent observations in relation to the values of the independent variables of the model.
2. A linear function of regressors
  (1) $$\nu_{i}=\alpha_{1}x_{i1}+\alpha_{1}x_{i2}+\cdots +\alpha_{1}x_{ik}+\gamma$$
3. A linearizing link function g(·) that converts the expectation of $y_{i}$, $\chi_{i}$ in $\nu_{i}$
  (2) $$g\left(\chi_{i}\right)=\nu_{i}=\alpha_{1}x_{i1}+\alpha_{1}x_{i2}+\cdots +\alpha_{1}x_{ik}+\gamma$$

#### 2.3.3. Random Forest

Random Forests (RF) are constituted by an ensemble of classification trees made through bootstrapping of the training dataset [24]. An important characteristic of RF is that the trees are poorly correlated with each other due to a randomization process of the features in the training phase. In fact, in the construction step of the trees, by means of an reiterate process, at each node, a subset of features is randomly selected. In general, RF have some characteristics that make them ideal in many machine learning analyses:

- They are ease to tune;
- There are only two different parameters to set: the number of trees *n* and *m* the number of features sampled to grow each leaf within a tree;
- They are little affected by the overfitting problem;
- They can evaluate the importance of each feature in the model during the training phase;
- By means of out-of-bag procedure, the Random Forest algorithm computes an unbiased estimate of the generalization error.

In the present work, we implemented a standard configuration in which each forest is composed by 1000 trees and m=f/3 where *f* represents the number of features used to train the model. The feature importance was evaluated through the mean decrease impurity. In the feature importance procedure, the RF algorithm measure the impurity decrease due to each variable by averaging over the whole forest. In our configuration node, impurity is measured by the residual sum of squares [24]. To achieve an accurate regression, RF should provide in the optimization phase a low correlation between residuals of differing regressor trees and a minimization of the prediction error function for the individual trees.

#### 2.3.4. Support Vector Machine

Support Vector Machine (SVM) is a machine learning technique that uses mathematical functions, called kernels, to translate data in a new hyperspace to simplify the representation of complicated patterns present in the data. The detailed description of SVM is reported in many works [25].

In the recent past, SVM has been implemented to solve regression problems showing several advantages over the traditional neural networks. In fact, SVM requires the setting of a few parameters, and it is robust to overfitting problems [26]. In the feature space, the regression equation can be expressed by:

(3) θ(x,w)=w·ψ(x)+z

where $x=x_{1},\cdots ,x_{N}$ are vector elements assumed to be statistically independent and identically distributed, w represents the vector of weights, ψ(x) is the feature function and *z* is a constant. In the regression problem, the SVM algorithm minimizes the following function:

(4) $$f=C\frac{1}{N}L_{\epsilon }\left(y,\theta \left(x,w\right)\right)+\frac{1}{2}\left\|W^{2}\right\|$$

with

(5) $$L_{\epsilon }\left(y,\theta \left(x,w\right)\right)=\left\{\begin{array}{cc} 0 & \mathrm{if}\;|y-\theta (x,w)|\leq \epsilon \\ |y-\theta (x,w)|-\epsilon & \mathrm{otherwise} \end{array}\right.$$

In Equation (4), *f* represents the empirical error, *C* quantifies the optimization between the empirical error and the model, *y* is the scalar real dependent variable and $L_{\epsilon }\left(y,\theta \left(x,w\right)\right)$ defines a loss function called ϵ-insensitive loss function [27]. Now, by introducing the Lagrangian multipliers λ and $\lambda^{*}$, the optimization problem is transformed into the dual problem. The input vectors $x_{i}$ with non-zero coefficients are called support vectors. Therefore, Equation (3) becomes:

(6) $$\theta \left(x,˘,{˘}^{*}\right)=\sum_{i=i}^{N}\left(\lambda_{i}-\lambda_{i}^{*}\right)K\left(x,x_{i}\right)+z$$

where $K(x,x_{i})$ represents the kernel function. The constant *z* is computed though the Karush–Kuhn–Tucker conditions [28]. In this work, we implemented a default configuration with a linear kernel.

#### 2.3.5. Feature Importance Procedure and Performance Metrics

For both GLM and SVM algorithms, the feature importance is estimated using the relationship between each predictor and the outcome. Specifically, we computed through $R^{2}$ the agreement of a model created by each feature to the outcome, thus obtaining a partial ranking. We adopted a 5-fold classification framework to further strengthen the robustness of our estimates and minimize overfitting issues. In this method, the initial dataset containing data of 21 Italian regions is randomly divided into 5 subsets without re-insertion: 5−1 subsets represent the training set, and the remaining part is used for validation. The described procedure is repeated *N* times in order to obtain *N* models with the relative performances. The average of the latter represents a reliable indicator of the model’s accuracy. In the feature importance procedure, we estimated a feature importance ranking by means of the 5-fold cross-validation model. In particular, for each cross-validation cycle, we assigned a weight to each feature according to its importance in the model measured by: (i) The impurity decrease for RF; (ii) $R^{2}$ statistic for GLM and SVM. We obtained an overall ranking by repeating the procedure N=500 times averaging over all repetitions. The data analysis procedure is summarized in Figure 2. To evaluate the performance of the three implemented models, we computed the coefficient of determination between the predicted values and actual values and the mean absolute percentage error (*MAPE*) [29]:

(7) $$MAPE=\frac{1}{n}\sum_{i=i}^{N}\left|\frac{A_{t}-F_{t}}{A_{t}}\right|$$

where $A_{t}$ is the actual value and $F_{t}$ is the forecast value. All the processing and statistical analyses were performed in R version 3.6.1 [30].

## 3. Results

### 3.1. Regression Performances

In Table 2, we reported some statistics for the nine independent features used in our models.

**Figure 3:**
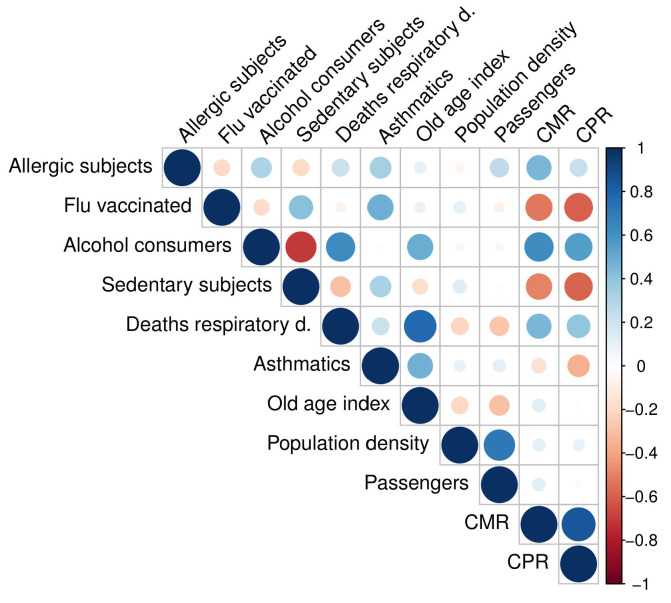
Correlation matrix for independent features, Crude Mortality Rate (CMR), and Crude Positivity Rate (CPR).

Firstly, we investigated the linear correlations [31] between each feature used in our study; see Figure 3.

**Figure 4:**
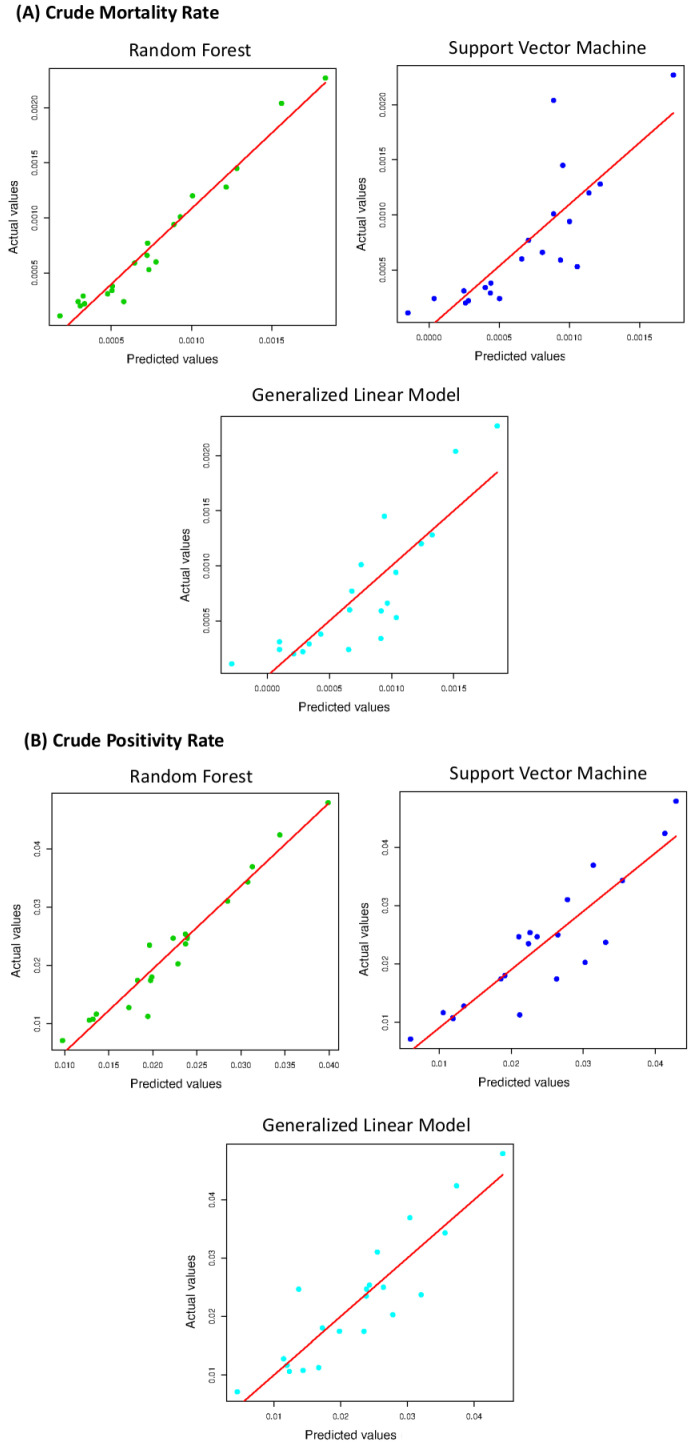
The agreement between the predicted values the actual values for CMR (panel **A**) and CPR (panel **B**).

We observed that the most positively or negatively correlated variables were the sedentary subjects and alcohol consumers (r=−0.70), deaths for respiratory diseases and the old age index (r=−0.77). Correlations ranging from 0.6 to 0.8 are generally considered moderate; therefore, we did not exclude any variable from the analysis. We evaluated MAPE and $R^{2}$ for the three implemented models. The agreement between the CPR predicted values and the CPR actual values and between the CMR predicted values and the CMR actual values are shown in Figure 4 for the three used algorithms.

The three models follow a positive linear trend. An overview of the regression performances is summarized in Table 3. RF appears to be the best performing algorithm.

The used models achieved forecasts with very strong correlations. In the CPR prediction, the Pearson correlation between RF and SVM is r=0.94; that between RF and GLM is r=0.92; and that between RF and SVM is r=0.94. Instead, for the CMR prediction, the Pearson correlation between RF and SVM is r=0.87; that between RF and GLM is r=0.90; and that between RF and SVM is r=0.93. We observed that the performances of the three different approaches differed by a few percentage points. The best-performing method was the RF algorithm both for the CPR (MAPE=0.17±0.02; Adjusted $R^{2}=0.96\pm 0.05$) and the CMR (MAPE=0.31±0.04; Adjusted $R^{2}=0.94\pm 0.05$) forecast, as shown in Table 3. Therefore, a non-linear model performs better than a linear model and SVM with a linear kernel, but the three investigated algorithms show a good prediction agreement.

### 3.2. Feature Importance

**Figure 5:**
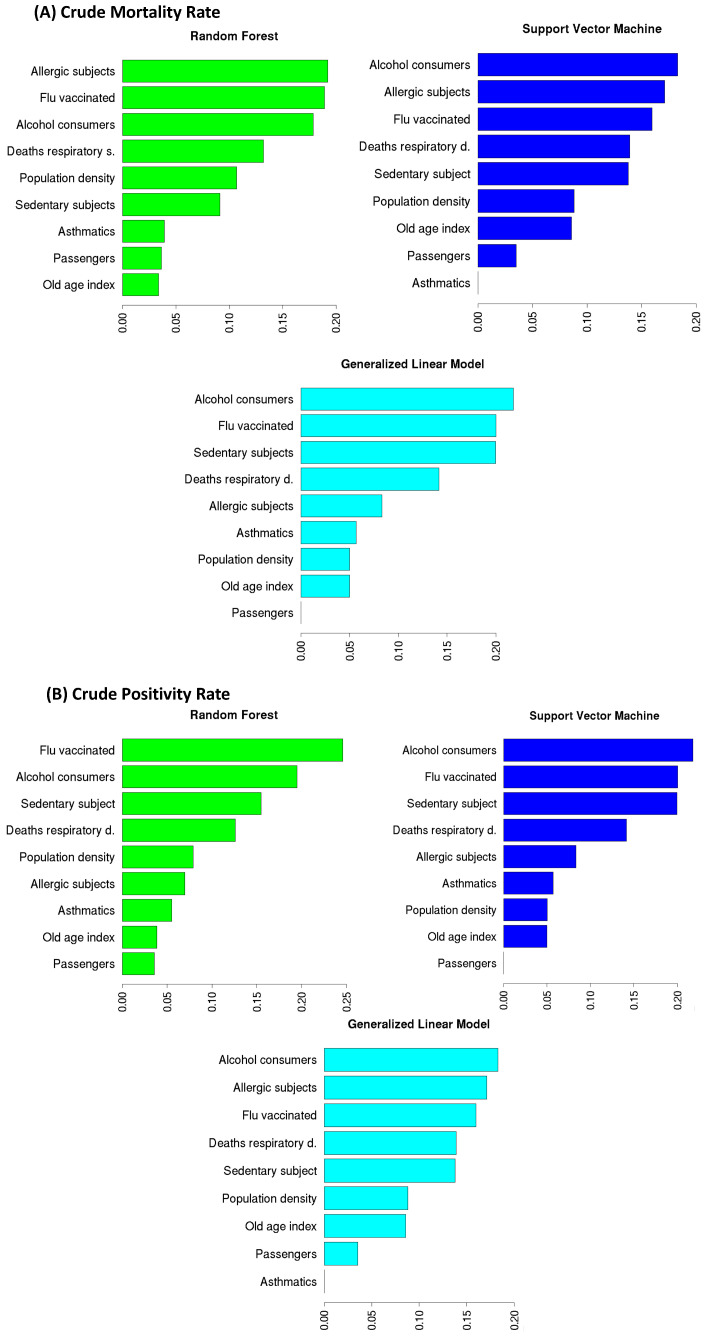
The feature importance produced by RF, SVM and GLM to predict CMR (panel **A**) and CPR (panel **B**).

Here, we investigated the most important features for the three implemented models to forecast CPR and CMR, as shown in Figure 5. Through a feature importance procedure based on a cross-validation technique, we observed that the three models in the CPR forecast had the same variables in the four most important places: Flu vaccinated, Alcohol consumers, Sedentary subject, and Respiratory deaths. Specifically, for GLM and SVM, the three top ranked features were the same: the most important variable in the CPR forecast was Alcohol consumers (22.8% and 21.8% is the percentage of importance for GLM and SVM, respectively), which was followed by Flu vaccinated (20% for both GLM and SVM) and Sedentary subjects (19% for GLM and 20% for SVM). For the RF model, the most important feature in predicting CPR was Flu vaccinated (24.6%) followed by Alcohol consumers (19.5%) and Sedentary subjects (15.5%). The three implemented models agreed on the fourth most important feature, namely Respiratory deaths (GLM 13.2%, SVM 14% and RF 12.6%). These four features gave the main contribution (about 74%) in the forecast models and had the highest linear correlation with CPR (see Figure 3). The three used algorithms are also quite in agreement on the four top rank features in the CMR prediction: Alcohol consumers (18.4% of importance for GLM and 18.3% for SVM, 17.9% for RF), Allergic subjects (17% of importance for both GLM and SVM, 19.2% for RF), Flu vaccinated (17% of importance for GLM, 16% for SVM and 18.9% for RF), and Respiratory deaths (12.9% of importance for GLM, 13.9% for SVM and 13.2% for RF). These results confirm the findings found for the CPR forecast except for the Allergic subjects feature, which replaced the Sedentary subjects feature.

## 4. Discussion

In this work, we compared three different forecasting algorithms—GLM, SVM, and RF—to predict the mortality and positivity ratios of the SARS-CoV-2 outbreak in Italy from 22 February to 22 November 2020. The use of three different models makes our results more robust; in particular, we started with GLM and then used SVM and RF as more complex and alternative models to the linear hypothesis. In our analysis, we divided the Italian territory into 21 regions and included in the model nine features (see Table 1) grouped into three macro categories (1) incidence of respiratory diseases, (2) lifestyle, and (3) mobility. Our results shows that in general, categories 1 and 2 influence the results more than category 3. It is interesting to note how the population density and the feature that in our model represents mobility (Passengers) are, as per expectations, strongly correlated with each other (r>0.7) but not very important in the model. In fact, these two factors, if taken into consideration separately, do not prove to be conducive to the establishment and spread of the virus. On the contrary, if they are considered jointly, they reveal a real condition of fragility (gatherings with distancing difficult) as reported in the literature [32].

**Figure 6:**
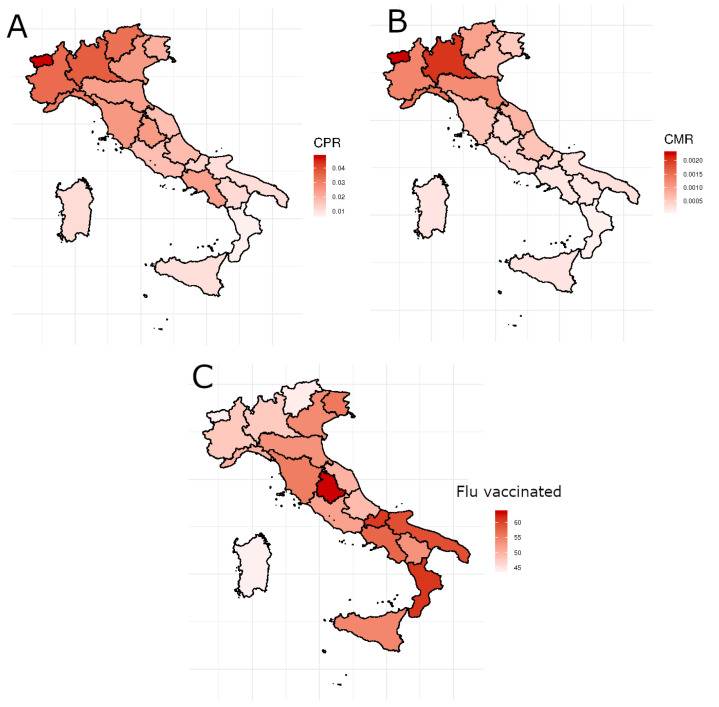
The geographical distribution in Italy of CPR (panel **A**), CMR (panel **B**) and the percentage of people over 65 years old vaccinated against the seasonal flu 2019 (panel **C**) in Italy.

Figure 6 shows the geographical distribution in Italy of CPR (panel **A**) and CMR (panel **B**) and the percentage of people over 65 vaccinated against seasonal flu 2019 (panel **C**). Considering that the deaths positively associated with SARS-CoV-2 in Italy have a negative gradient from north to south and vaccinations have a higher percentage in the south than in the north, it is clear that the correlation with vaccinations is negative (correlation equal to −0.59 and −0.53 for CPR and CMR respectively), so there are fewer deaths and positives where the number of over 65 vaccinated is higher. In addition, subjects who have a sedentary lifestyle appear to be less at risk of contracting COVID-19 (Pearson coefficient equal to −0.58). Probably for their lifestyle, they have less chance of contracting the disease. Instead, the positive correlation between the feature Alcohol consumers and CPR (0.56) and CMR (0.62) highlights that subjects who abuse alcohol seem particularly vulnerable to COVID-19.

Previous works confirm our results. Several studies found that flu vaccination is also linked with COVID-19 severity and mortality, playing some sort of protective role against severe symptoms.

Marín-Hernández et al. [33] reported a link between the higher uptake of influenza vaccination and lower deaths from COVID-19 in Italy. Tayar et al. [34] in a study implemented on a population of 30,774 subjects assessed an effectiveness of 88.9% for flue vaccination against severe or mortal effects of COVID-19. Huang et al. [35] found that the influenza vaccine may slightly protect people from COVID-19 infection. It has long been known how lung pathophysiologies can be aggravated by alcohol abuse, which could therefore increase both susceptibility and severity to COVID-19 [36], so it should come as no surprise that the feature Alcohol consumers is highly important in our models. Muhammad et al. reported that six genes linked with the severity of COVID-19 disease (CCR2, DPP9, HSPA1L, TYK2, OAS1, ACE2, and TMPRSS2) were also upregulated in the brain tissue of the population of habitual alcoholics.

There is controversial evidence of the association between asthma and allergies and the risk of adverse clinical outcomes of COVID-19. Some works [37,38,39] reported that asthma is not a risk factor for SARS-CoV-2 infection, as confirmed by our models. It is known that the COVID-19 virus triggers an overwhelming host immune response. Larson et al. [40] demonstrate that the genetic pathways underlying the susceptibility to allergic diseases are protective against COVID-19. Wu et al. [41] through a study on 1,169,441 subjects found that patients who died from COVID-19 were not at risk of asthma, either.

### Limitations and Strengths

It is worth mentioning that our work is not an exhaustive study on the SARS-CoV-2 outbreak in Italy because we neglected many possible causes such as the different ways of dealing with the pandemic by the Italian local health system. It is very difficult to choose a group of factors that can explain such a complex problem as the spread of COVID-19 in Italy in an exhaustive way. Our model is also an approximation of the problem. Then, even the regional scale is too broad to understand the precise dynamics of the territory, but the availability of the data did not allow us to choose a finer geographical scale.

The use of machine learning can weaken these limitations thanks to its ability to extract salient information from the data without making prior assumptions. Furthermore, the use of COVID-19 severity data available up to November 2020 avoided inserting an additional bias in our model due to the start of the vaccination campaign. Our model and the obtained results could be a strategic way to better understand the mechanisms of diffusion and physiology of this disease.

## 5. Conclusions

The causes of COVID-19 spread in Italy in the the first wave of the pandemic are difficult to explain even because the real onset date is not clear. A mathematical model that explains this diffusion should be very complex and take several factors into account. In this paper, we have tried to identify nine features related to health and lifestyle risks that could be connected to COVID-19 spread. We used a data-driven approach based on machine learning techniques to predict and explain the degree of severity of COVID-19 in Italy through these factors. Furthermore, an in-depth study on the feature importance allowed us to quantify which factors had the greatest impact on diffusion. Our study could be replicated and validated on a European scale also including data from subjects vaccinated against the 2020 seasonal flu to better understand the clinical and physiological meaning of the pandemic.

## Figures and Tables

**Table 1:** Summary table with some information of independent features used in our model.

Independent Feature	Explanation
Allergic subjects	percentage of people affected by chronic allergic diseases in 2016 in each Italian region [16].
Flu vaccinated	percentage of people over 65 years old vaccinated against the seasonal flu in each Italian region in 2019 [17].
Sedentary subjects	percentage of subjects, in each Italian region, that do not engage in any physical activity in their free time, nor do they do heavy work calculated from 2015 to 2018 [18].
Deaths respiratory system	number of deaths due to diseases of the respiratory system per 100,000 inhabitants in each Italian region in 2016 [19].
Asthmatics	percentage of subjects suffering from chronic bronchitis and bronchial asthma in each Italian region in 2019 [19].
Alcohol consumers	percentage of subjects who claim they have a high daily alcohol consumption in each Italian region calculated from 2015 to 2018 [19].
Old-age index	ratio between the population aged 65 years and over and that under 15 in each Italian region in 2019 [19].
Population density	population density expressed in inhabitants per square kilometer in each Italian region in 2019.
Passenger	data collected by each Italian national airport about the passengers who departed from or landed at that airport in 2018.

**Table 2:** Summary table with some statistics of independent features.

Independent Feature	Mean	Standard Deviation	Median	25th Percentile	75th Percentile
Allergic subjects	11.1%	1.3%	10.7%	9.9%	11.3%
Flu vaccinated	52.1%	6.6%	53.3%	49.1%	55.7%
Sedentary subjects	32.0%	12.3%	28.7%	23.8%	38.3%
Deaths respiratory system	83.0	15.0	77.2	72.9	96.5
Asthmatics	5.9%	1.0%	6.0%	5.5%	6.4%
Alcohol consumers	3.3%	1.3%	3.6%	2.4%	4.4%
Old-age index	175.2	30.5	177.8	155.5	195.9
Population density	177.7	113.5	162.0	79.0	206.0
Passenger	8,841,969	1,412,266	3,193,386	223,436	8,893,672

**Table 3:** Summary table of regression performance measures obtained through the implemented models. MAPE: Mean Absolute Percentage Error; SD: Standard deviation.

Predicted Values	Regression Models	MAPE (±SD)	Adjusted $R^{2}$ (±SD)
Crude Positivity Rate	Random Forest	0.17±0.02	0.96±0.05
	Support Vector Machine	0.18±0.03	0.80±0.05
	Generalized Linear Model	0.19±0.02	0.81±0.05
Crude Mortality Rate	Random Forest	0.31±0.04	0.94±0.05
	Support Vector Machine	0.44±0.05	0.66±0.05
	Generalized Linear Model	0.59±0.05	0.72±0.05

## Data Availability

Data and R codes used to perform the analysis are available upon request.
